# A critical review on the availability of substandard and falsified medicines online: Incidence, challenges and perspectives

**DOI:** 10.1177/23992026221074548

**Published:** 2022-02-15

**Authors:** Jamee Ahmed, Laura Modica de Mohac, Tim K Mackey, Bahijja Tolulope Raimi-Abraham

**Affiliations:** 1 Institute of Pharmaceutical Sciences, School of Cancer and Pharmaceutical Science, King’s College London, London, UK; 2 Department of Anesthesiology and Division of Infectious Diseases and Global Public Health, School of Medicine, UC San Diego, La Jolla, CA, USA

**Keywords:** Substandard medicines, falsified medicines, fake medicines, online, Internet

## Abstract

Simultaneous expansion of the Internet and increased globalisation of the pharmaceutical industry have meant medication can be accessed transnationally from both legal and illicit sources. This has coincided with the rise of substandard and falsified medicines (SFMs) online. These products fail to meet regulatory or quality standards and/or are constituted with substandard ingredients, causing undesired pharmacological effects, including possible injury and death. This review aimed to identify original research studies that examined characteristics of SFM online sales, attitudes towards purchasing medicines online and strategies to address this drug safety challenge. Keywords of ‘Substandard’ and ‘Falsified’/‘Counterfeit’ and ‘Medicines’/‘Drugs’ and ‘Online’/‘Internet’ were searched using Web of Knowledge and PubMed databases. Resulting literature, which satisfied the study’s inclusion criteria, was included in the review, and the findings from each paper were assessed. From an initial 185 literature articles, 7 were eligible according to the inclusion criteria to be reviewed. These articles identified studies testing SFMs purchased online, surveys of attitudes and knowledge about SFMs online, and website content analysis to detect illegal online sales. Challenges identified were lack of knowledge and awareness among consumers and physicians, in addition to the use of direct-to-consumer-advertising, via Internet platforms and social media, providing easy access to SFMs. Despite this, medicine authentication technology, website verification approaches and new detection methods were identified as potential solutions specific to online SFM sales. To address online sales of SFMs, more robust research, greater awareness/educational programmes, analytical detection methods and more stringent online global governance are required.

## Introduction

High-quality, safe and efficacious medicines are fundamental in decreasing global morbidity and mortality.^1^ Technological advancements and Internet access, with appropriate regulation, have enabled users access to safe, legitimate products. Unfortunately, this has coincided with the rise in the illicit substandard and falsified medicine (SFM) trade.^1^

The majority of literature published in the last two decades utilises definitions established in 1992 by the World Health Organization (WHO).^1^ According to WHO, ‘substandard medicines’ are genuine products which unachieved quality standards.^2^ Official pharmacopoeia is used to derive these standards, which help ensure safety, establishing common quality requirements. WHO defines ‘falsified medicines’ as products misrepresented deliberately in terms of identity, formulation, and/or source.^2^ As there is no universally established definition for SFMs, other synonyms, such as counterfeit, are commonly used.^3^ In this review, WHO’s definitions for SFMs, as previously stated, will be used.

Reports indicate 10% of medicines worldwide could be SFMs; however, this is difficult to quantify.^4,5^ This threat is not new, as cases of falsified cinchona bark and quinine, respectively, were reported as early as the 1600s and 1800s.^6^ Recent SFM cases include falsified diazepam containing haloperidol in Congo, resulting in dystonic reactions in 930 individuals in 2014 (including 11 fatalities),^7^ falsified bevacizumab was seized in the United States in 2014 and emerging later in Uganda during 2017,^8^ and the United Kingdom’s Medicines and Healthcare products Regulatory Agency (MHRA) identified more than 34,000 falsified COVID-19 products seized worldwide in 2020.^9^

SFMs may have the correct active pharmaceutical ingredient (API) but at incorrect doses, false API or no API. This also relates to excipients, which may be present in inaccurate concentrations or absent. Consequently, drug products may not produce the desired pharmacological effect. Packaging/labelling may also be implicated, deceiving consumers of authenticity, source and ingredients.^2^

SFMs are more prevalent in low- and middle-income countries (LMICs), where numerous supply chains run in parallel, causing variation in storage, time and supervision.^10^ As of 2017, the SFM market in LMICs is estimated to be worth between US$10 billion and US$200 billion, with a median value of US$31.5 billion.^11^ These values are likely to be underestimated due to difficulty in monitoring multijurisdictional criminal activity.^12^

SFMs are not exclusive to LMICs as incidences increased from 1997, after the erectile dysfunction medication, sildenafil, was launched in the United States. Authorities in numerous regions, including Asia and Europe, noticed the emergence of illegal erectile dysfunction drug trade online.^13^

Despite SFMs being a worldwide problem, legal implications vary among countries. In the majority of high-income countries (HICs), SFMs are less likely to infiltrate the supply chain, due to laws regarding medicine manufacture, distribution and dispensing. However, LMICs have a greater prevalence due to insufficient healthcare systems, weak regulations and outdated laws.^14^ In addition, increased law enforcement against the illicit drugs trade, including cocaine and heroin, meant criminals altering their operations to target the SFM market.^12^ Moreover, the involvement of the Internet means illicit actors worldwide can participate in trading SFMs.

The World Wide Web is evolving, and cybercrime is becoming a greater threat, with now over half the world online.^10^ In 2015, the US Federal Bureau of Investigation stated a US$1,070,711,522 cybercrime cost, which included economic impacts linked to the illegitimate Internet pharmaceutical market.^15^ Motivations for criminals to utilise online platforms include customer reach, accessibility and anonymity. For the consumer, online purchases are convenient, accessible around the clock, typically more economical and confidential as users are not required to share personal information. Furthermore, consumers may use online sources to obtain prescription-only medication (POM), without a prescription.^16^

Driving growth in this market are the increasing numbers of people who are utilising the Internet to obtain health information, as attitudes of taking responsibility for their health have changed, along with reduced trust in authorities.^17^ Consumers may obtain medication from online sources, though these channels may be illegitimate.^12^ This is an area of concern as approximately 72% of Internet users in the United States search online for health information and medical advice, whereas a third use the Internet to self-diagnose.^18^

The US Food and Drug Administration (FDA) approximates 23% of Internet users have brought POM online.^18^ Research indicates that millions of Americans purchase medication online; however, it is rarely reported to healthcare professionals.^15^ Pharmacists have reported concerns about patients purchasing medicines online, due to safety risks, complicating medication management and issues during counselling. Selling POM without a prescription or using questionnaires to sell POM instead of prescriptions is an illegal act committed by vendors. Other illegal activities include displaying misleading, false or imprecise statements and selling/exporting medication without authorisation. Estimates from the National Association of Boards of Pharmacy (NABP) in 2014 suggest roughly 4% of online drug traders operate legally.^12^ Purchasing from online sources is risky, as products quality and safety are questionable and available without clinical oversight. SFM procurement may occur on social media platforms, such as Facebook and Twitter, which have high user traffic and enable SFMs to be ‘pushed’ to users.^12^ Given almost 80% of active Internet users visit social media sites regularly, this further expands SFMs reach.^18^ Other marketing tactics utilised to reach a wide demographic online, include affiliate networks, email spam and website adverts. In addition, purchasers may fall victim to cybersecurity risks, including computer viruses/malware/spyware infection, financial fraud and data phishing.^10^

Hence, this critical review investigates SFMs availability online, attitudes towards purchasing medicines online, and strategies to overcome this growing patient safety challenge. This review aims to identify original research articles characterising the risks posed by SFMs while focusing on the incidences, challenges and perspectives of this crucial global health challenge.

## Method

### Literature identification

A non-systematic literature review was conducted using keyword combinations associated with SFMs and online sales searched in reference sources with large science/health literature databases. The search terms were ‘substandard’ and ‘falsified/counterfeit’ and ‘medicines/drugs’ and ‘online/Internet’. These keywords were selected based on WHO definitions for SFMs and similarly validated keywords commonly used in published literature. In addition, ‘online/Internet’ were searched as this study focuses on e-commerce. Web of Knowledge (https://www.webofknowledge.com/) was a reference source used. Keywords were inputted into the search field and searched using the ‘Topic’ setting. Using PubMed (https://pubmed.ncbi.nlm.nih.gov/), keywords were queried using the ‘Search PubMed’ field using basic search settings. Both settings allowed keywords to be searched in titles, abstracts and author keywords found in Web of Knowledge and PubMed (MEDLINE) databases. Search outcomes were filtered to remove duplicate entries.

### Literature refinement

**Figure 1.**
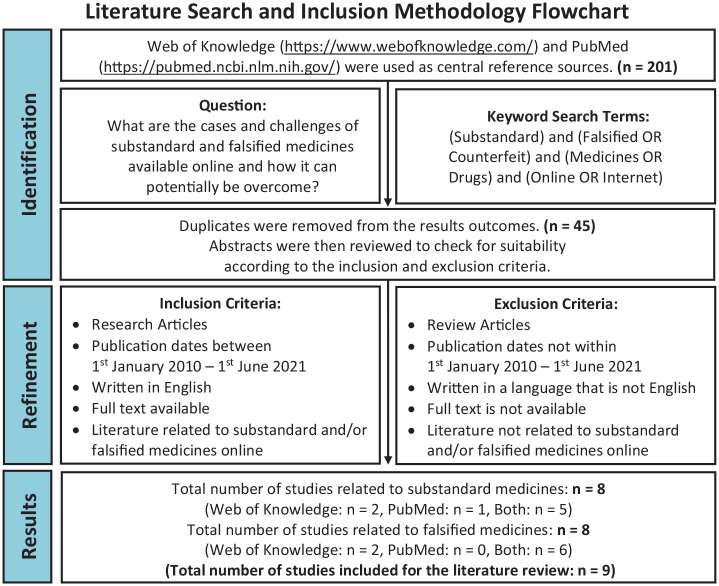
Flowchart depicting the methodology used in this review and the number of articles generated in each phase. Using a combination of keyword search terms, an initial 201 articles were obtained from Web of Knowledge and PubMed. After removing duplicates, the abstracts from the remaining 45 articles were reviewed according to the inclusion and exclusion criteria. A total of nine articles were eligible to be included in the literature review.

Results were refined according to inclusion and exclusion criteria, as summarised in Figure 1. Inclusion criteria included research articles related to SFMs online written in English, with full text available and published between 1 January 2010 and 1 June 2021. Exclusion criteria included non-original research articles, articles not written in English and outside desired publication dates. A 10-year timeframe was selected as this study aimed to identify recent SFM cases sold online while also trying to identify contemporary challenges and potential solutions. Unrelated articles and those without full texts were also not reviewed. Literature was filtered to only obtain research articles. The exclusion criteria removed reviews, commentaries, conference papers and news reports. Abstracts were screened to determine whether they reported results from original studies involving the detection and characterisation of SFMs sold online. During this refinement, article sources, study methodology and relation to SFMs were noted. Two authors independently reviewed all study papers for inclusion and exclusion criteria for this study.

## Results

### Literature search results

The identification phase generated 201 articles, and removing duplicates yielded 45 unique articles. The initial search outcomes identified two studies outside the desired publication dates (4.4%) and 24 review articles (53.3%). The majority were written in English (97.8%) and one was in French (2.2%). After screening abstracts, nine articles were eligible for inclusion in the literature review. The nine articles were conducted in six countries, including China (*n* = 1), Hungary (*n* = 1), Japan (*n* = 3), Sweden (*n* = 2), United Arab Emirates (UAE; *n* = 1) and the United States (*n* = 1). Five articles related to pharmaceutical analysis (55.6%), three involved surveys (33.3%) and two utilised website characteristic analysis (22.2%).

Pharmaceutical analysis articles highlighted SFMs for conditions, such as diabetes and hypertension, giving insight into conditions being treated online. Surveys investigated SFM knowledge, which can assist in improving awareness and helping professionals deal with SFM users. Website analysis explored user traffic to identify regions where there was a demand for medication. These data could be used to enforce greater measures in these countries to protect consumers.

## Pharmaceutical analysis

Pharmaceutical analysis articles (*n* = 5) detected drug classes online of dietary supplements (*n* = 1), Chinese patent medicines (*n* = 1), furosemide (*n* = 1), atorvastatin calcium (*n* = 1), metformin (*n* = 1) and erectile dysfunction (*n* = 1). A variety of techniques were utilised, including liquid chromatography (*n* = 3), Raman spectrophotometry (*n* = 2), infrared spectrophotometry (*n* = 1), ultraviolet-visible spectrophotometry (*n* = 1), powder X-ray diffraction (PXRD; *n* = 1), X-ray computed tomography (CT; *n* = 1), principal component analysis (PCA; *n* = 1) and testing of disintegration (*n* = 1), dissolution (*n* = 3), friability (*n* = 1) and hardness (*n* = 1). Key findings from these articles are summarised in Table 1.

**Table 1.** Summary of the Key Findings From the Three Articles Which Performed Pharmaceutical Analysis of the Contents of the Medicines Advertised on Online Websites (Adapted from Previous Works^19–23^).

Authors	Medication	Testing method		Results					
Guo et al.^19^	Sulfonylureas in dietary supplements and Chinese patent medicines purchased from online and physical markets in China	Novel skeleton-type molecularly imprinted column for online 2D liquid chromatography		Glibenclamide was identified in one sample out of five batches at approximately 0.97 mg/g					
Ashames et al.^20^	Furosemide purchased from online and physical markets in the United Arab Emirates (UAE)	According to BP (2018)		*UAE market furosemide*			*Online furosemide*		
			Disintegration Test	Pass (all tablet disintegrated completely within <30 min)			Pass (all tablets disintegrated completely within <30 min)		
			Dissolution Test	Pass (>80% of the API dissolved within 45–60 min)			Pass (>80% of the API dissolved within 45–60 min)		
			Friability Test	Pass (0.6%)			Pass (0.3%)		
			Hardness Test	Pass (6.1 kp)			Pass (7.1 kp)		
			Identification Test	Pass (confirmed by ultraviolet-visible spectrophotometer)			Pass (confirmed by ultraviolet-visible spectrophotometer)		
			Assay Test	Pass (100.2% ± 0.7%)			Fail (91.0% ± 0.8%)		
Fukami et al.^21^	Atorvastatin calcium from four Japanese-language websites			*J1 (Aztor)*	*J2 (Lipitor)*	*J3 (Lipiget)*	*J4 (Atorlip)*	*J5 (Lipvas)*	*J6 (Lipitor)*
		Received date		31 January 2012	15 December 2011	15 December 2011	20 December 2011	20 December 2011	15 December 2011
		Expiry date		1 July 2014	1 January 2014	1 May 2014	1 November 2013	1 June 2012	1 January 2013
		Countries of manufacture		India	Thailand	Pakistan	India	Unknown	Germany
		Tablet appearance		Brown and circular	White, oval and with markings	White, oval and with markings	White and oval	White and oval	White, oval and with markings
		Packaging when received		Bubble wrapped and opened	Opened	Were as shown online	Were as shown online	Opened	Were as shown online
		Package leaflet		Yes	No	No	Yes	Yes	No
		According to JP (16th edition)	Assay Test	Pass (100.6%)	Pass (100.8%)	Pass (100.2%)	Pass (98.4%)	Pass (99.3%)	Pass (103.0%)
			Dissolution	Pass (>80% dissolved after 15 min). J3 had noticeably slower dissolution compared to the rest.					
		Raman spectroscopy		Characteristic peaks identified around 1450, 1150 and 900 cm^−1^.					
		X-ray CT		–	–	Coarse particles and aggregates	–	–	–
Authors	Medication (drug class)	Testing method		Results					
Zhu et al.^23^	Metformin tablets obtained via the Internet in Japan (strengths of 500, 750 and 1000 mg)	Visual observations		• A 500-mg tablet sample originated from Singapore; however, the package insert and press-through package sheet were written in Japanese. The package insert also contained the lot number and expiration date, which is not common in Japan. This sample arrived without a box. • Another 500 mg sample indicated the dose as 500 mg on its box packaging; however, the insert described it as 850 mg. Measuring the ingredient contents determined that the actual contents were 500 mg. • 16 samples provided in a box were written in English. One sample had a box written in English and Thai. The press-through package sheet was written in Japanese (*n* = 1), Chinese (*n* = 1) and English (*n* = 9). All samples in bottles and aluminium–plastic composite film package were written in English (*n* = 8 and *n* = 4, respectively). The packaging insert was provided with 12 of 40 samples and was written in Japanese (*n* = 1), English (*n* = 9), Chinese (*n* = 1) and Thai (*n* = 1). • India (*n* = 18), UK (*n* = 6), France (*n* = 2), Thailand (*n* = 2), Japan (*n* = 1) and Canada (*n* = 1) were listed as manufacturing countries, while New Zealand (*n* = 1) was noted as a distribution country. This information was absent in 10 samples. • Samples were shipped from Thailand (*n* = 18), Taiwan (*n* = 1), US (*n* = 5), India (*n* = 3), Hong Kong (*n* = 3) and Malaysia (*n* = 3). One sample had parcel posts from both Singapore and Hong Kong. • Sample 16, 28 and 40 had a physical crack, yellow stain and blue stain, respectively.					
		Authenticity		• Only 7 of the 40 samples were indicated as genuine, as responses to the questionnaire were received from the manufacturer/distributor. Despite reminders being sent, no response was received; the authenticity of the other 33 products is unknown.					
		Evaluation of website details		• Based on the details required on websites, 6 of the 24 websites listed all necessary details. According to the requirements of the Act on Securing Quality, Efficacy and Safety of Products Including Pharmaceuticals and Medical Devices, 22 websites did not adhere fully to the recommendations.					
		Quantification of metformin		• Concentration in 4 samples (three 500 mg and one sustained-release tablet) and was outside the limits. • Three 500 mg tablets had values of 94.5 (± 1.5%), 93.6 (± 5.7%) and 107.0 (± 2.8%). • A sustained-release tablet had a value of 116.7 (±16.5%).					
		Content Uniformity Testing		• A total of 35 samples, out of 40 samples, passed the content uniformity test. The 5 samples that did not pass had acceptance values between 16.6 and 54.9.					
		Dissolution Test		• Dissolution testing passed in 38 samples. A 500-mg sustained-release tablet and a 1000-mg extended-release tablet failed dissolution testing, with dissolution rates of 68.6 (±3.3%) at 3 h and 87.0 (±6.3%) at 6 h, respectively.					
Authors	Medication (drug class)	Testing method	Sample	Results					
Sanada et al.^22^	Tadalafil, vardenafil and sildenafil tablets purchased from the Internet	Raman spectroscopy and principal component analysis (PCA)	33 Tadalafil tablets (1 standard, 9 genuine and 23 falsified)	• 12 of the 23 falsified tablets presented peaks in the Raman spectra with the same wavenumbers, but different intensities, as the standard tablet. • 1 falsified tablet had decreasing spectra intensity with increasing wavenumber, in addition to approximately no peaks being exhibited. In the remaining 10 falsified tablets, no peaks were evident due to fluorescence and the Raman spectra produced was flat. • PCA indicated that the standard and genuine tablets were similar, as the points on the plot were very close together. PCA also noted that 10 falsified tablets were not similar to the standard tablets, while the other 13 falsified tablets were close to the standard in principal component-1 (87%) but not in principal component-2 (12%).					
			23 vardenafil tablets (1 standard, 9 genuine and 13 falsified)	• A falsified sample had 2 peaks present with the standard tablet at 510 and 630 cm^−1^, but with different intensities compared to the standard sample. A different falsified tablet had multiple peaks which were significantly different from the standard tablet at 1240, 1400 and 1520–1590 cm^−1^. These 2 falsified tablets displayed similar spectra to that of sildenafil, suggesting these falsified vardenafil tablets contained sildenafil. • 11 falsified tablets produced fluorescence and therefore no peak was displayed, and the spectrum was flat. • The PCA showed 4 authentic tablets were close to the standard. • However, 2 falsified samples had no significant peaks compared to the standard in principal component-1 (94%). The remaining falsified tablets showed similarity in principal component-1 and/or principal component-2 (5%). • A falsified sample had almost identical Raman spectra to the other falsified tablets; however, the PCA plot showed that this sample was closer to the standard sample.					
			23 sildenafil tablets (1 standard, 4 genuine and 18 falsified)	• A small peak was seen in the spectra for 17 out of the 18 falsified tablets, at approximately 1000 cm^-1^, which was absent in the standard or genuine sample spectrums. • In addition, 2 falsified tablets displayed no peaks and had decreasing intensity on the spectrums with increasing wavenumber. Difficultly was seen in identifying some falsified tablets visually as spectra for the falsified and standard tablets were comparable. • PCA found the 4 genuine tablets were similar to the standard sample. Also, 16 falsified tablets out of 18 were shown to be similar to the standard and genuine tablets in principal component-1 (94%) and/or principal component-2 (5%). The PCA plot also showed that a falsified tablet was comparable to the standard tablet, despite the Raman spectra being similar to the other falsified tablets.					

BP: British Pharmacopoeia; API: active pharmaceutical ingredient; JP: Japanese pharmacopoeia; CT: computed tomography; PCA: principal component analysis.

Guo et al. developed a novel skeleton-type molecularly imprinted column and tested five batches of dietary supplements and Chinese patent medicines purchased online and from physical markets.^19^ Results identified glibenclamide in one sample at approximately 0.97 mg/g; however, the source of the products was unclear.^19^

Another study evaluated the chemical and physical qualities of furosemide tablets purchased illegally online and from the UAE, according to the British Pharmacopoeia (BP).^20^ Both tablets passed weight variation, disintegration, dissolution, friability, hardness and identification tests.^20^ However, chemical analysis, via ultraviolet-visible spectroscopy, highlighted online furosemide was below acceptable limits (91.0% + 0.8%), indicating it is substandard.^20^

Fukami et al. investigated the quality of six atorvastatin calcium tablets (J1-Aztor, J2-Lipitor, J3-Lipiget, J4-Atorlip, J5-Lipvas and J6-Lipitor) from four Japanese-language websites.^21^ The country of manufacture includes India, Germany, Pakistan and Thailand, while J5-Lipvas’ origin was unknown.^21^ Comparing packaging and product appearance with website images, three tablets were shown, including shape, colour and size.^21^ J5-Lipvas appeared most questionable due to lack of information on packaging, unclear manufacturing country and expiry date of 6 months from receipt date.^21^ Tablets were assayed to establish dissolution profile and API contents, according to the Japanese Pharmacopoeia (JP). Both dissolution and API assay satisfied the JP; however, J3-Lipiget had a noticeably slower dissolution.^21^ Raman spectroscopy identified atorvastatin calcium’s characteristic peaks in all tablets around 1450, 1150 and 900 cm^−1^.^21^ PXRD and infrared spectroscopy identified J3-Lipiget mainly contained lactose and starch. Examining X-ray CT inner morphology of J3-Lipiget and compared to Lipitor control, multiple aggregates and heterogeneous particles were seen, whereas Lipitor had fine uniform particles.^21^

Sanada et al. used Raman scattering spectrometry and PCA for the identification of falsified samples from online-purchased tadalafil, vardenafil and sildenafil. For tadalafil, 52.2% of falsified tablets, compared to the standard, presented spectra peaks with the same wavenumbers, but different intensities.^22^ The falsified tablets exhibited no peaks and the spectra were flat.^22^ PCA indicated the standard and genuine tablets were similar and that 13 falsified tablets were close to the standard in principal component-1 only.^22^ For vardenafil, a falsified sample had two common peaks with the standard (510 and 630 cm^−1^), but differing intensities.^22^ Two falsified tablets displayed similar spectra to sildenafil, suggesting its presence. Eleven falsified tablets produced fluorescence and therefore displayed a flat spectrum.^22^ Two falsified samples had no significant peaks compared to the standard in principal component-1.^22^ The remaining falsified tablets showed similarity in principal component-1 and/or principal component-2.^22^ For sildenafil, a small peak was seen in 94.4% of falsified tablets, at approximately 1000 cm^-1^, which was absent in the standard or genuine sample spectrums.^22^ Falsified and standard generated comparable spectra. PCA indicated the genuine and standard tablets were similar. Sixteen falsified sildenafil tablets were similar to the standard/genuine in principal component-1 and/or principal component-2, despite the Raman spectra for the falsified tablets being similar to the other falsified tablets.^22^

Zhu et al. investigated the authenticity and quality of 40 metformin tablets purchased online.^23^ When obtaining samples, prescriptions were not requested. Visual inspections detected three tablets with abnormalities, including blue and yellow stains and a crack.^23^ Carton and packaging insert dosage discrepancy was noted in one sample, which stated 500 mg and 850 mg, respectively. Analysis established the content was 500 mg.^23^ Inconsistency was present in packaging languages against the leaflet and declaring manufacturing/distribution countries.^23^ Authenticity was based on questionnaire responses from each manufacturer/distributor, covering sample information (e.g. API content, doses). Seven products were deemed to be genuine; however, the authenticity was unclear for the remainder as no response was received.^23^ Based on the US Pharmacopoeia (USP), samples were tested for metformin content, content uniformity and dissolution testing. Four samples failed content testing, where three samples were 500 mg tablets and one sustained-release tablet.^23^ Five samples failed content uniformity testing with values ranging from 16.6 to 54.9.^23^ A 500-mg sustained-release tablet and a 1000-mg extended-release tablet failed dissolution testing.^23^ Findings determined that 7 metformin samples were authentic, while the remaining 33 were questionable.

## Surveys

Key findings from the three survey articles, which were performed in Sweden (*n* = 2) and the UAE (*n* = 1), are summarised in Table 2. Surveys examined consumers’ attitudes towards purchasing medication online (*n* = 2), and SFM knowledge/experience among general practitioners (GPs) and emergency physicians (EPs; *n* = 1).

**Table 2.** Summary of the Key Findings From the Three Articles Which Performed Surveys to Identify Participants Knowledge and Attitudes Towards SFMs Online (Adapted From Previous Works^17,20,24^).

Authors	Survey topic	Sample	Survey method	Key findings	
Funestrand et al.^17^	Substandard and falsified medicine knowledge and experience	100 general practitioners and 100 emergency practitioners in Sweden	Online survey with anonymous answers to 9 questions (a mixture of open-ended and multiple-choice questions)	Knowledge of the term	• 157 practitioners (78.5%) were aware of the term ‘illegal and falsified medicines’ before this survey. • From those who were familiar with the term, the most popular source was media (87.3%; including daily newspaper (70.1%), medical journals (61.3%) and TV (55.5%)). • Patient-related events (31.9%) and work colleagues (30.6%) were other major sources.
				Suspecting patients using SFMs	• 38 EPs (38%) and 35 GPs (35%) had suspected patients taking SFMs. • EPs mentioned that patients’ medical history (55.3%) and patients’ symptoms (44.7%) suggest patients taking SFMs. GPs said their main clues suspecting SFM use came from medical history (77.1%) over symptoms (22.9%). • Medication most commonly suspected include opioids/benzodiazepines (14) and anabolic steroids (7). Others mentioned include medication for erectile dysfunction (2) and hepatitis C (1), health products (1) and antibiotics (1). • 11 comments from practitioners referred to online ordering.
				Unfamiliar medicines	• 137 (68.5%) practitioners had come across medication which was unfamiliar to them. • 78 practitioners (56.9%) said these medications were foreign pharmaceuticals. • The most frequent medicine types were antibiotics (24.1%), analgesics (19.0%), cardiovascular medication (15.3%) and hypnotics/sedatives (9.5%). Herbal remedies/health products (including vitamins) represented 8.8%. Other and unknown made up 28.5% and 2.9%, respectively. • Patients sourced products from abroad (75.5%) or via the Internet (16.1%). • Practitioners’ reaction is to try to identify the medication (40.9%), counsel/inform patients of the risks (30.7%) or discourage SFM use (16.8%).
				Need for more knowledge	• 157 practitioners (78.5%) stated a greater need for more knowledge about SFMs. • The most popular method to obtain this knowledge is through written material, such as newspaper articles (69.4%), followed by lectures (48.4%) and online education (39.5%).
Lundin and Liu^24^	Attitudes of the public on medicine consumption	155 members of the public in Sweden	Online survey with 14 questions (a mixture of open-ended, single-choice and multiple-choice questions)	• 81% would obtain prescription-only medication through doctors with the healthcare system. • 11% of respondents, however, would purchase prescription-only medication without consulting a healthcare professional and use abroad countries or online to source these medications. • 63% of participants failed to recognise the European Union official logo displayed on legally operating online pharmacies.	
Authors	Survey topic	Sample	Survey method	Key findings	
Ashames et al.^20^	Public perceptions about buying medication from online sources	528 United Arab Emirates (UAE) residents aged 18 years and above	Survey with 13 questions (divided into three sections: 1. Sociodemographic data 2. Practice and experience of medication e-commerce 3. Knowledge and behaviour assessment related to medication e-commerce)	Sociodemographic data	• 397 participants (75%) were female, while 131 participants (25%) were male. • 408 participants (77%) were educated to university level or higher. • 262 participants (50%) were between 18 and 25 years, the most popular age group.
				Practice and experience of medication e-commerce	• 9.7% had previously purchased medication online. • Over-the-counter/non-prescription medication accounted for the most popular medicines obtained from online sources (78%). Prescription medications accounted for 6%, while the remaining 16% was responsible for both prescription and over-the-counter/non-prescription medicines. • In descending order, the most frequent therapeutic area/drug class purchased using the Internet in the UAE are food supplements/vitamins (41%), obesity/overweight (31%), pain (16%), chronic disease (8%), cancer (2%) and infections (2%). • Low prices and non-availability were the main reasons medicines were being purchased online in the UAE, accounting for 43% each. Other reasons include wide product choice (10%), no need for a prescription (2%) and other (2%). • When asked whether the online medication improved the participant’s health, over half (51%) stated that there was little improvement, while 14% indicated no improvement. However, 35% of participants noticed a remarkable improvement. • Participants expressed that 45% of them would consider ordering medicines online in the future, while 39% and 16% conveyed that they are not sure and will not, respectively.
				Knowledge and behaviour assessment related to medication e-commerce	• 321 individuals (61%) were uncertain about the quality of medication brought online, while 26 participants (5%) believed it to be of high quality. Individuals who thought the quality of medicines obtained from online sources was reasonable or very poor/fake accounted for 12% and 22%, respectively. • Almost half (45%) stated they would never buy medication online in the future. However, 106 people (20%) said that they would. The remaining 35% were not sure if they would. • 78% expressed that they were unaware of the law regarding the illegal nature of medication e-commerce in the UAE, while 22% were aware.

SFM: substandard and falsified medicine.

Results found 78.5% of physicians were aware of the term ‘illegal and falsified medicines’, while 36.5% suspected users.^17^ The percentage of GPs and EPs who encountered a patient they suspected to have taken SFMs was 35% and 38%, respectively.^17^ Participants stated the three most frequent medications were benzodiazepines/opioids (*n* = 14), anabolic steroids (*n* = 7), and erectile dysfunction medication (*n* = 2).^17^ Foreign medicines accounted for 56.9%, where most were sourced from abroad (75.5%) or online (16.1%).^17^ The most common foreign medicines were antibiotics (24.1%) and cardiovascular medications (13.9%).^17^

When asked about participants’ response to patients they believed were highly suspicious of taking SFMs, 64.5% would try to influence patients’ opinion, whereas 5.0% stated possibly reporting to the health authority.^17^ Seeking further information was recommended to patients by only 6.5%, primarily by contacting other healthcare professionals.^17^ Most physicians (78.5%) stated a need for greater understanding about SFMs, and 69.4% indicated using written material as the preferred method, for instance, newspaper articles.^17^

Another article examined public perceptions of consuming medication in Sweden.^24^ Results indicated 81% obtain POMs via their doctor.^24^ However, 11% considered buying from unsafe sources, such as the Internet, bypassing contact with physicians.^24^ Furthermore, 63% did not recognise the online pharmacy verification logo, required by the European Union (EU) used to indicate the legitimacy of online pharmacies.^24^ Many respondents suggested this logo was important for safety; however, some expressed concerns over misuse on illegitimate websites to appear authentic and law-abiding.^24^

The final study examined public perception of medicine e-commerce in the UAE, which indicated that 9.7% purchased medicines online, where 78% were non-prescription.^20^ E-commerce motivations were mostly due to cost-effectiveness (43%) or unavailability in-store (43%).^20^ Food supplements (41%) and weight loss products (31%) were responsible for the majority of medication being sourced online.^20^ Only 4.9% of participants thought the medicines were of high quality, whereas 22% believed it was very poor quality or fake.^20^ Despite 60.8% of participants stating uncertainty about the quality of the medicines, 45% would reorder in the future whereas 16% stated they would not.^20^ A concern reported was 77.8% were unaware of UAE’s laws on medication e-commerce.^20^

## Website characteristic analysis

Mackey and Liang evaluated the accessibility of direct-to-consumer advertising (DTCA) through social media for an illicit online pharmacy. False adverts were created for illicit, non-prescription drugs and posted on Facebook, Twitter, Google+ and MySpace. The number of visiting users and locations was examined between 24 September 2011 and 24 July 2012.^18^ The other study aimed to identify safety and professionalism indicators in 136 online pharmacies. The authors mimicked how potential customers would identify online pharmacies by searching on the popular search engine Google. Websites were then assessed at 6- to 10-month intervals between February 2008 and February 2012. An online database, LegitScript, determined pharmacies characteristics, including location, prescription requirement and legitimacy verification.^25^ Table 3 summarises the findings from these articles.

**Table 3.** Summary of the Key Findings From the Two Articles Which Analysed Websites (Adapted From Previous Works^18,25^).

Authors	Aim	Method	Parameters	Key findings				
Mackey and Liang^18^	To evaluate the accessibility of direct-to-consumer advertising via social media, for an illicit no prescription online pharmacy	Fictional advertisements for no prescription drugs available online were posted on Facebook, Twitter, Google+ and MySpace. Using Web search analytics and commercially available tools and services, key parameters were analysed.	• Number of visitors • Visitor location	• All social media content remained available, despite advertising potentially illegal content and breaching terms and conditions. • Only the Google+ account was suspended for undisclosed reasons, approximately 4 weeks post the website being active. • All the fictitious adverts generated a total of 4107 visits from 2795 unique visitors over 10 months. Twitter generated the most traffic volume and the most unique visitors. User traffic originated from 18 countries. The United States generated more than half (54.0%) of traffic. The Top 4 countries with the highest traffic represented the majority (95.7%) of all traffic generated. • Locations of users clicking on the fictitious advertisement on the four social media platforms:				
				*Countries (economic classification)*	*Social media platforms, n (%)*			
					*Facebook*	*Twitter*	*MySpace*	*Total*
				The United States of America (high income)	702 (34.2)	818 (37.7)	650 (30.0)	2170 (54.3)
				China (upper-middle income/emerging market)	240 (23.1)	412 (39.7)	387 (37.2)	1039 (26.0)
				The United Kingdom (high income)	105 (8.8)	145 (9.6)	106 (8.2)	356 (8.9)
				Russia Federation (upper-middle income/emerging market)	67 (5.6)	76 (5.0)	117 (9.0)	260 (6.5)
				Unknown	38 (3.2)	12 (0.8)	7 (0.5)	57 (1.4)
				Germany (high income)	7 (0.6)	9 (0.6)	8 (0.6)	24 (0.6)
				Japan (high income)	5 (0.4)	15 (1.0)	4 (0.3)	24 (0.6)
				The Netherlands (high income)	4 (0.3)	5 (0.3)	5 (0.4)	14 (0.4)
				France (high income)	4 (0.3)	4 (0.3)	5 (0.4)	12 (0.3)
				Ukraine (lower-middle income/emerging market)	2 (0.2)	2 (0.1)	2 (0.2)	6 (0.2)
				Israel (high income)	2 (0.2)	2 (0.1)	2 (0.2)	6 (0.2)
				The Czech Republic (high income)	2 (0.2)	2 (0.1)	2 (0.2)	6 (0.2)
				Republic of Moldova (lower-middle income)	3 (0.3)	2 (0.1)	2 (0.2)	7 (0.2)
				Canada (high income)	3 (0.3)	1 (0.1)	1 (0.1)	5 (0.1)
				Sweden (high income)	2 (0.2)	0 (0.0)	0 (0.0)	2 (0.1)
				Australia (high income)	1 (0.1)	1 (0.1)	1 (0.1)	3 (0.1)
				Taiwan (high income)	1 (0.1)	1 (0.1)	1 (0.1)	3 (0.1)
				Romania (upper-middle income)	1 (0.1)	1 (0.1)	1 (0.1)	3 (0.1)
				Republic of Korea (high income)	0 (0.0)	2 (0.1)	0 (0.0)	2 (0.1)
Authors	Aim	Method	Parameters	Key findings				
Fittler et al.^25^	Identify safety and professional indicators in online pharmacies	136 online pharmacy websites were identified from a consumer point of view and were assessed over 4 years using the LegitScript database	• Longevity • Time of continuous operation • Geographical location • Displayed contact information • Prescription requirement • Medical information exchange • Legitimacy verification	• Frequency of active online pharmacies decreased over time. A total of 23 (16.9%) of 136 online pharmacies stopped operating within a year, while 67 (49.3%) were available at the end of the study. Almost one-fifth (22.8%) were temporarily inaccessible, while 41.2% were operating continuously throughout the 4-year window • Websites not providing contact information accounted for 22.8%. However, all necessary contact details were seen in 43.4% of pharmacies. Majority (75.0%) of the websites displayed their telephone numbers; however, only 46.3% provided physical addresses. These addresses were in Europe (18.4%), North America (14.0%), Pacific (3.7%), Caribbean (2.9%) and elsewhere (7.3%). No addresses were seen in 53.7%. • The domain registration area (according to IP address) and declared physical locations did not correlate with most websites. Only 23.5% of domains were in the same continent as the declared postal location. In addition, 9.6% servers were within the same countries borders. The IP addresses indicated the most popular domains were the United States (50.7%), the United Kingdom (10.3%), Russia (6.6%), Panama (5.1%), Israel (3.7%), Canada (2.9%), the Netherlands (2.9%), New Zealand (2.9%) and elsewhere (14.7%). • Prescription-only medications were available on 120 websites (88.2%), but only 9 websites (6.6%) requested a valid prescription before a purchase could be made. Some pharmacies (11.8%) specialised in over-the-counter medication exclusively, while 52.9% exclusively sold generic medication. Websites only selling brand name medicines accounted for 19.1%. Online pharmacies selling more than 10 different active pharmaceutical ingredients were responsible for 81.6% of the sample. Websites selling one active ingredient represented 9.6%. • Exchange of medical information was ineffective. Questionnaires were used in 84 (61.8%) of pharmacies to obtain patients health information. However, medical information from patients was not required on 52 online pharmacies (38.2%) before buying. Online consultation, via email, online chat and telephone, was available on 66 (48.5%) of websites. Information about the products was not seen on the majority (92.6%) of websites, and this was also true of the patient information leaflet (76.5%). • According to LegitScript, many Internet pharmacies (44.1%) in this study were identified as rogue. One website (0.7%) was not yet verified, and 23 (16.9%) were unapproved. The remaining 52 (38.2%) online pharmacies were not in the LegitScript database. No approved legitimate online pharmacies were identified from the 136 websites. From the long-lived pharmacies in this study, 30.4% were unapproved and 64.3% were rogue. From the 67 (49.3%) online pharmacies available at the end of the study, 8 pharmacy-related seals were identified on 11 (16.4%) websites. This included Canadian International Pharmacy Association and PharmaCheck seals (three websites each), TrustedRx and PharmacyChecker logo (two websites each) and a Registered Internet Pharmacy (UK) (1 website). Majority (83.6%) of the active pharmacies did not have any professional or pharmaceutical logo. LegitScript or Verified Internet Pharmacy Practice Site verification logos were not present on any website in this study.				

Insufficient regulation exists on social media platforms, as posts remained available despite possibly violating the terms and conditions. Only the Google+ account was suspended for undisclosed reasons. Facebook, Twitter and MySpace remained linked to fictitious advertisements and generated 4107 visits from 2795 unique visitors over 10 months.^18^ Findings indicate users’ traffic from 18 countries, with 54.3% coming from the United States. The second highest was China (26.0%), while another emerging market, Russia was fourth (6.5%). With the United Kingdom ranking third (8.9%), these four countries represented 95.7% of user traffic.^18^

Fittler, Bősze and Botz highlighted 16.9% of pharmacies stopped operating within a year, and within 4 years, 50.7% were not accessible.^25^ Also, 22.8% of online pharmacies ceased operations at least once and then revived during the study.^25^ Contact details, such as telephone number or postal address, were not displayed in 22.8%, whereas 43.4% provided both.^25^ Telephone numbers were present on 75% of websites, while locations were seen in 46.3%.^25^ From pharmacies indicating their address, 18.4% was in Europe, 14.0% was in North America, 3.8% was in the Pacific and 2.9% was in the Caribbean.^25^ Just 23.5% of domains, according to Internet protocol (IP) address, correlated to the same continent stated on the website.^25^ Based on IP addresses, the top locations were the United States, the United Kingdom and Russia, representing 50.7%, 10.3% and 6.6%, respectively.^25^ Up to 88.2% of the 136 pharmacies offered various POMs, while 6.6% requested prescriptions.^25^ Similarly, 38.2% of pharmacies did not request any medical information from users, while 61.7% used questionnaires.^25^ Eight pharmacy-related seals were present on 16.4% of websites, including the likes of the Canadian International Pharmacy Association (37.5%), PharmaCheck (37.5%) and TrustedRx (25.0%).^25^ LegitScript described websites as rogue (44.1%), unapproved (16.9%) or unverified (0.7%).^25^

## Discussion

Pharmaceutical analyses noted the identification of glibenclamide in dietary supplements/Chinese patent medicines.^19^ Another study found online-purchased furosemide failed BP testing for API content with a value of 91.0%.^20^ Fukami et al.^21^ discovered that one sample of atorvastatin calcium, from four Japanese-language websites, had coarse particles/aggregates and noticeably slower dissolution. Sanada et al.^22^ showed Raman spectra and PCA identified all falsified tadalafil tablets, and falsified vardenafil tablets could be identified by Raman spectra, while the PCA score was similar to the standard tablet. Discrepancies identified in online-purchased metformin included the country of origin and packaging language, dosage difference in the packaging/insert and physical abnormalities. In addition, samples were noted to fail the content uniformity testing, limits for quantification and dissolution testing.^23^

Surveys highlighted that physicians had encountered medication unfamiliar to them and stated a need for SFM knowledge.^17^ Another survey identified individuals who would purchase POM, without consulting a professional, and a large proportion did not recognise the EU legal online pharmacy logo.^24^ Ashames et al. noted less than 10% of the UAE;s public had previously purchased medication online, of which over-the-counter/non-prescription medication accounted for a large proportion. The main motivations for medication e-commerce were non-availability in stores and cost-effectiveness. Almost 80% expressed they were unaware of the e-commerce laws in the UAE.^20^

Social media content remained available online, despite advertising potentially illegal content. User traffic originated from 18 countries, with the United States representing the largest proportion.^18^ Fittler et al. determined the number of active pharmacies decreased over time and that over a fifth did not provide contact information. Domain registration area did not align with the declared physical locations on most websites. POMs were available on approximately 90% of websites; however, only 6.6% requested a prescription. Despite many pharmacies in this study being classified as unapproved or rogue, they were still available online.^25^

Guo et al. identified glibenclamide in dietary supplements/Chinese patent medicines. Substandard and falsified glibenclamide has been fatal in many countries.^26^ Glibenclamides’ inexpensiveness allows it to falsify anti-diabetic drugs such as repaglinide which is comparatively more expensive.^27^ In 2009, 6 times the standard dosage of glibenclamide caused the hospitalisation of nine individuals and two fatalities in China.^28^ Findings are consistent with the literature, suggesting issues with substandard and falsified glibenclamide and other diabetes medication.^29^

Despite furosemide tablets containing correct API in this review, literature has noted cases where furosemide was replaced with zopiclone.^30^ Consequently, patients will experience zopiclone’s sedative effect and not furosemides diuretic effect.^31^ Another study identified furosemide injections being sold on websites classed as ‘not recommended’ by the NABP.^32^ Despite different dosage forms, this highlights furosemide availability from questionable websites.

An atorvastatin calcium sample had a slower dissolution rate; therefore, bioavailability and efficacy would be less.^33^ As it is used in treating hypercholesterolemia, incomplete/slow dissolution can cause cardiovascular issues.^34^ Since this sample’s dissolution rate satisfied the JP, dissolution-related adverse effects are unlikely. Not all online drugs meet dissolution standards, as seen in a study that found 17 falsified vardenafil tablets.^35^ Counterfeit vardenafil contained sildenafil or tadalafil and even some products containing vardenafil failed to meet the dissolution specification.^35^

Raman spectroscopy benefits over infrared spectroscopy were noted, including measurements without pre-treatment and rapid measurements, which could explain the increased use in recent years.^21,35^ However, Raman devices cannot detect substandard medication and are better suited for identifying falsified medications.^36^ LMICs may benefit from handheld Raman and near-infrared spectroscopy due to low cost, ease of use and measurement speed with no sample preparation or electrical supply.^37^

Sanada et al.^22^ utilised Raman spectroscopy and PCA to identify tadalafil tablets. Falsified vardenafil was identifiable with Raman spectra.^22^ The majority of the falsified sildenafil tablets had similar spectra peaks to the standard.^22^ Findings suggest falsified tadalafil from online sources could be detected via Raman spectroscopy and PCA; however, other techniques are required to determine the nature of vardenafil and sildenafil. The standards for all three medications had peaks between 510 cm^−1^ and 630 cm^−1^, which is most likely due to the titanium oxide film coating, included for photoprotection and colouring. Criminals may consider this is unnecessary and not include it in the formulation. The authors also noted sildenafil in falsified vardenafil tablets, which may be due to cross-contamination during manufacturing or deliberately included to increase the effect of the treatment.

Zhu et al. established online sellers did not confirm prescriptions for metformin, a POM in Japan. In addition, a lack of questionnaire responses from the majority of manufacturers/distributors meant sample authenticity could not be determined.^23^ Substandard and falsified diabetic medication have been identified worldwide.^26^ The presence of Japanese metformin online suggests an unauthorised supply chain exists.^23^ Subsequently, product quality may be compromised due to incorrect transportation and storage conditions. Another concern includes non-Japanese language on the packaging and inserts of 98% of samples.^23^ Patients may not comprehend the information presented, potentially leading to adverse side effects. Zhu et al.,^23^ however, did discover some samples failed the content uniformity and/or quantitative analysis, which can cause inconsistent or insufficient therapeutic effects, and lead to serious complications.

SFM attitudes from this review were consistent with Law and Youmans,^38^ noting 59.3% of pharmacists in California believed it is a serious issue; however, 60% had no SFM experience. Reporting in Sweden was lower than in California, as 52.4% of pharmacists would report the incident to the FDA, corporate headquarters or board of pharmacy.^38^ This suggests SFM education is more available in the United States; however, both countries should encourage reporting to authorities and educating consumers.

Website analysis indicated simplicity and inexpensiveness to market SFMs, via social media DTCA, which is coherent with literature examining FDA shortage drugs and highest-grossing drugs of 2009.^32,39^ The ability to broadcast in many countries violates DTCA legislation, excluding New Zealand and the United States.^18^ Twitter was most used for DTCA, consistent with another study stating the greater presence of recalled medicines sold on Twitter than Facebook.^40^ Other social media platforms used for drug trading include Instagram, Snapchat, Slideshare and Flickr.^41,42^

Insufficient product information was displayed in 92.7% of pharmacies analysed, and 76.5% had incomplete information leaflets.^25^ Lack of product information was also noted on illegitimate online pharmacies.^43^ Without information, such as dosage and ingredients, consumers are at risk. Even websites providing safety information may be unregulated.^44^

China and Russia had the second and fourth greatest user traffic, which also have a high frequency of online pharmacies.^18,45^ The United States and the United Kingdom complete the Top four, both having high-profile SFM incidences.^12^ For example, in 2012, counterfeit avastin was detected in clinics across the United States.^46^ The highest traffic was generated from the United States, which is consistent with published literature for being the largest pharmaceutical market.^12^ Results show that 23.5% of domains correlated to the same continent as the locations on the website, lower than a study that found 55% corresponding.^25,47^ This decrease could be due to greater use of masking domain location to authorities.^48^

As English-speaking countries are targeted by approximately 80% of Internet pharmacies, it is unsurprising 61.0% of domains originated from these countries.^25,49^ Canadian and US IP addresses have been used for opioid sales, while registered addresses were in Italy and Pakistan, respectively.^50^ Falsified contact information could entice consumers. For instance, Canadian pharmacy appeal has been noted, due to costs and trustworthiness based on Canadian safety seals.^51,52^ However, these websites may not be in Canada or accredited by seal providers. In addition, 61.8% of online pharmacies used questionnaires; however, this has been described as unsuitable for health assessment and provides consumers with a false sense of assurance.^25,48^ There is no evidence that questionnaires are reviewed by professionals, putting consumers at risk.

The majority of online pharmacies offered POM without prescriptions and lacked information, which is a negative safety indicator. Despite this, online pharmacies’ longevity was associated with illegitimate activities. However, ‘revival’ websites accounted for 22.8% of online pharmacies.^25^ The authors followed up websites every 6–10 months; however, visiting more frequently will provide greater accuracy.^25^ Motivations for websites temporarily becoming inactive could include avoidance of discontented customers/law enforcement. Given illegal online pharmacies have the capabilities of concealing contact information, it is expected this opportunity would be taken, as seen in 22.8%.^25^

Despite not purchasing from websites, relatively small sample size, and limited study duration, this literature review highlights warning signs, such as the POM sale without prescription and disparity between domain location and address, which may indicate SFM sale. Monitoring and analysing these indicators could help devise strategies to reduce SFM e-commerce.

## Policy implications of review findings and recommendations

Regulatory authorities, social media companies and Internet providers need to take greater action to reduce SFMs online. With suboptimal regulations, ease of launching online pharmacies and DTCA, unscrupulous personnel produce and sell SFMs online while benefiting from inadequate laws, increasing e-commerce and anonymity.

In a WHO member state survey, legislation on online pharmacy regulation was absent in 66% of countries.^53^ Even countries with specific laws in place may be insufficient or outdated. For example, the US federal law introduced the Ryan Haight Online Pharmacy Consumer Protection Act (RHA) in 2008, aimed at reducing Internet medication sales by prohibiting controlled substance sales.^54^ However, the scope of RHA is limited to controlled substances listed by the Drug Enforcement Administration, excluding other medications sold online, and is limited to US traders, meaning sellers in other countries can continue to sell to the United States.^55^ Despite the RHA, online pharmacies are still selling ‘no prescription’ medication.^55^ This highlights the need for an updated global approach to legislation that involves many stakeholders to protect Internet users, opposed to country-specific regulations.

International and multidisciplinary collaborations will help protect patients by identifying and shutting down online SFM sellers. International Criminal Police Organisation (INTERPOL) announced a global programme in collaboration with 29 of the largest pharmaceutical companies to fight pharmaceutical crime. Other key stakeholders involved include law enforcement, pharmaceutical/wholesaler industries, Internet providers, credit card companies, health regulators and customs agencies.^55^ INTERPOL has previously proved to be successful. Operation Pangea VI led to 9.8 million medications (worth US$41 million) being seized, more than 9000 websites being shut down and 58 individuals being under investigation or arrested.^55^ A similar model with the involvement of key stakeholders necessary may be needed to tackle SFMs online.

Active keyword monitoring may identify traders, leading to account/website closures and prosecution while protecting patients. Targeting keywords associated with high-risk or common medications could be lifesaving. For example, contraceptive and insulin products have been sold online by questionable sources.^42,56^

Websites could utilise a verification scheme, as seen with the EU common logo displayed on websites meeting specific authenticity requirements in the EU member states. Clicking on the logo redirects users to the national list of legislation-compliant pharmacies.^49^ However, as expressed by participants in the study by Lundin and Liu,^24^ accreditation seals may be falsified by illegal websites and misrepresented as accredited. A promising solution is NABP’s ‘.pharmacy’ generic top-level domain (gTLD) name, which distinguishes between different website types.^43,57^ The ‘.pharmacy’ gTLD is issued to websites complying with NABP criteria and is more difficult to forge than verification seals.^49^ With NABP estimating 97% of online pharmacies are not compliant with pharmacy laws/standards, greater efforts are needed to combat this issue.^53^

Given education and training varies by role, institution, and country, the worldwide inclusion of SFMs online would better equip professionals to recognise and report. This also provides them with the knowledge to pass onto patients, improving awareness. In a review of pharmacy curriculum in two Asian and six sub-Saharan African countries, SFM training was only mentioned in one country.^58^ Better training should be developed, especially in LMICs where SFMs are prevalent, for example, USP’s collaboration with Nigerian universities on expanding pharmacists’ curriculum.^58^ However, this should also encapsulate the SFMs market online.

Improving awareness would help limit SFMs spread and prevent harm. In a US FDA survey, 23% of adults admitted to purchasing POM online, despite 29% lacking assurance in purchasing safe medication.^53^ With online pharmacies using DTCA strategies, which could disseminate misleading information about the benefits and/or risks of the SFMs, and the culture of self-diagnosing/self-prescribing through the Internet, greater consumer knowledge is required. Consumers may self-diagnose/self-prescribe without seeking professional advice, leading to purchasing unnecessary medicines, medicines with contraindications or abuse potential/danger to health even if the source is authentic. Moreover, the quality, safety or effectiveness of the medication cannot be guaranteed, potentially consuming SFMs. SFM awareness could be raised via government initiatives, for example, following a UK public survey, the MHRA recognised 79% of participants were unaware of SFMs.^16^ Consequently, the MHRA is developing a website with advice on distinguishing legal online traders.^16^ Alternatively, company-led social media campaigns may be an effective method. For instance, Pfizer developed its ‘Counterfeiters are Smart. You can be Smarter’ project to raise awareness about counterfeit medicines via YouTube.^28^ Social media has proven to be effective as a health intervention as seen with targeted initiatives for smoking cessation and weight loss.^53^

Social media use is now omnipresent and has made it an opportune marketplace for SFM sellers. Social media platforms should work to eliminate SFM sellers from these platforms. For example, Facebook and Instagram began blocking opioid-related hashtag searches and blocking users’ accounts in April 2018. Despite this, studies have shown this to be ineffective as sellers continue to operate, possibly by altering their hashtag use.^54^ Tackling SFMs sold via social media could save millions of lives, and in the case of opioids, improve the US opioid crisis. Similarly, fentanyl, an opioid-based analgesic POM which has been counterfeited and introduced into the US supply chain, is sold via Twitter. A study highlighted significant amounts of fentanyl-related tweets related to sales. Given the high potency and lethal nature of fentanyl, the presence of online sellers is alarming and lead to a rise in fentanyl-related overdoses, seizures and fatalities.^48^

To combat SFMs, efficient law enforcement, ample regulatory efforts and increased awareness are necessary.

## Limitations of the study

This reviews methodology could be optimised to retrieve more articles. Inclusion criteria restricted literature to those published between January 2010 and June 2020. Similarly, only Web of Knowledge and PubMed reference sources were used. Using others, such as Google Scholar, or ScienceDirect, may increase the number of articles. To obtain more articles, using synonyms or different keywords could be beneficial, for example, using ‘fake’ instead of ‘substandard’/‘falsified’. The literature obtained in this study may not represent the true SFMs’ scope.

The authors of two studies did not include copies of the surveys completed by participants, which may contain close-ended questions or limit answers to a few options.^20,24^ As a result, unanticipated information by the authors may be missed. In addition, some terms used in the survey could be interpreted differently. For example, ‘quality’ was used by Ashames et al.;^20^ however, it may have several meanings to participants, and the authors did not define it. These could have skewed the results in this literature review.

The literature obtained mainly focused on tablets. Further research is needed into other dosage forms, such as creams and suspensions. For example, a study in Nigeria had identified substandard/falsified ketoconazole creams.^59^ Alternatively, including specific dosage forms in the search terms may help identify related articles. Greater research into other dosage forms can provide further insight into vulnerable medications/patients.

## Conclusion

SFMs availability online is a global challenge difficult to combat. This article reviewed research from the last decade and identified cases of online SFMs, including atorvastatin calcium and furosemide. Some obstacles discovered are lack of education among consumers and physicians, as well as DTCA. Addressing these will result in greater awareness and healthcare professionals being better equipped to deal with SFM users. Strategies to help overcome SFMs, include pharmacy-specific gTLD, which allow consumers to differentiate between illegitimate and legitimate websites. Due to difficulty in falsifying, authorities could make it a requirement for online pharmacies and make consumers aware of this. Without improved awareness, greater monitoring and tougher global governance, online SFMs may continue to be an issue.

Future research could modify this study’s methodology to generate additional literature which provides greater detail on current research. Alternatively, researchers may investigate high-risk medications or therapeutic areas, which can lead to implementing patient-protecting measures. For example, due to differences between the minimum therapeutic and toxicity levels being closer in narrow therapeutic index medicines, SFMs could be fatal. Studies could develop detection methods for APIs and harmful compounds with improved accuracy. The development of cost-effective techniques will enable more sample testing, especially in LMICs where SFMs are more prevalent, leading to further identification and saving lives worldwide.

## Expert opinion and concluding remarks

There is a need to protect online consumers from two main aspects: their health, by ensuring the seller is genuine, and their cybersecurity, to ensure consumers are not victims to risks such as financial fraud and data phishing. This literature review has provided insight into the availability of SFMs online and has highlighted solutions that could be implemented in multiple areas to address the associated challenges. For example, greater awareness of SFMs online could improve through collaborative campaigns between national authorities and government agencies. Creating and/or updating legislation to prevent and prosecute illegal fake medicine traders online may be difficult to implement due to several factors including national law enforcement, the technology available and monitoring personnel in a rapidly developing Internet. Strategies to overcome SFMs online are hindered by various factors, including lack of international collaboration, financial resources and lax legislation and enforcement. Lack of international collaboration impedes the harmonisation of legislation, whereas financial resources will be beneficial to many countries, especially LMICs when it comes to monitoring suspicious online activity and creating greater awareness. The lack of awareness and knowledge among patients and healthcare professionals is a major hurdle identified in the research that must be addressed. Education on the dangers of SFMs and how to identify them online plays a key role in saving lives and preventing harm. This knowledge could be distributed by universities, health authorities, medical/pharmaceutical societies and/or pharmaceutical companies.

The focus of future research should shift to investigate the types of consumers purchasing medications online (i.e. age, socioeconomic background, etc.), different platforms used to purchase these products and the products purchased. Findings from this work would be useful to create solutions to deter consumers from making questionable purchases or inform them of the risks available. Future research could also focus on examining how new and existing technologies can be utilised to help protect patients from online SFMs. Blockchain is currently being investigated as a specific tool that can help secure supply chains and reduce the impact of SFMs. With the convenience of e-commerce and the increasing culture of self-diagnosing/self-prescribing, more people are purchasing medicines and other health products online. SFM sellers already exploit this demand and, with the help of the Internet, it allows them to remain anonymous and reach a large audience across the world. If these sellers’ websites or accounts get shut down or suspended, they can create more with relative ease. This makes it difficult for consumers to distinguish legitimate sources from fake. In addition, these criminals are smart, and they find loopholes and flaws in existing systems and technologies, to use for their advantage for profit. For example, online pharmacy accreditation seals were introduced to safeguard patients from deceptive sellers; however, this has been utilised by SFM sellers to entice users to visit their websites by exploiting the consumers’ trust, as seen in this literature review. Taking the above into consideration, unfortunately, one could also assume a correlation with the number of cases and fatalities associated with SFMs online to increase.

Greater safeguarding measures such as pharmacy-specific gTLDs and educational programmes could be implemented, as these are difficult for criminals to fake and have a wide audience reach. Pharmacy-specific gTLD names are an interesting area with great promise. The use of this methodology, to verify the authenticity of sellers online, could increase patient confidence by indicating that the site is complying with appropriate legislation. As discussed, ‘.pharmacy’ gTLD is regulated by the NABP which is an independent and non-profit organisation. Application of similar approaches globally with language considerations could create a safer global climate for online consumers.

With the recent COVID-19 pandemic, the pharmaceutical industry has begun investigating treatment with new and existing medications for its potential in fighting the SARS-CoV-2 virus. Criminals have also profited from this by selling SFMs through online platforms. Given the dangers this poses, not only for the treatment of COVID-19 but for other ailments too, SFMs online are a significant threat to health and well-being. As highlighted in this literature review, there are significant challenges in combating this concern. Overcoming these challenges will have positive real-world outcomes which will help safeguard patients from fake medicines, as well as prevent criminal enterprises from expanding and profiting. Changes in consumer practices, education and online safety may result from this research, which provides greater insight into the latest trends and activities in this ever-evolving area. With advances in technology, greater use of online platforms and the demand from patients to obtain medication online, this area must be addressed, and action is needed to be taken sooner than later.
